# Feasibility of assessing quality of care at the end of life in two cluster trials using an after-death approach with multiple assessments

**DOI:** 10.1186/1472-684X-13-36

**Published:** 2014-07-15

**Authors:** Emily West, Vittoria Romoli, Silvia Di Leo, Irene J Higginson, Guido Miccinesi, Massimo Costantini

**Affiliations:** 1 EMGO + Institute for Health and Care Research-Vrije Universiteit Medisch Centrum, Van der Boechorststraat 7, Amsterdam, 1081 BT, The Netherlands; 2 Regional Palliative Care Network, IRCCS AOU San Martino-IST, Genoa, Italy; 3 Department of Palliative Care, Policy and Rehabilitation-Cicely Saunders Institute, Kings College London, London, UK; 4 Clinical and Descriptive Epidemiology Unit, ISPO Institute for the Study and Prevention of Cancer, Florence, Italy; 5 Palliative Care Unit, IRCCS Arcispedale S. Maria Nuova, Reggio Emilia, Italy

**Keywords:** Retrospective study, End of life care, Methodological study, Feasibility study, Quality of health care, Cancer

## Abstract

**Background:**

In 2009 two randomised cluster trials took place to assess the introduction of the Italian Version of the Liverpool Care Pathway in hospitals and hospices. Before and after data were gathered. The primary aim of this study is to evaluate the feasibility of using a combination of assessment methods aimed at different proxy respondents to create a means of measuring quality of care at the end of life. We also aim to explore whether there are differences in response to this approach between the hospice and hospital inpatient settings.

**Methods:**

A retrospective design was used. Eligible deaths were traced through death registries, and proxies were used to give information. Four procedures of assessment were used to measure different dimensions. Feasibility was assessed through compliance and adherence to the study instruments, and measured against standards derived from previous after-death studies. The proxy caregiver’s rating of the study tools was also measured, to gauge feasibility and effectiveness. All consecutive cancer deaths that occurred in the study period were eligible. In both trials, deaths were excluded if the patient was a relative of hospital/hospice staff. 145 patients were recruited from the Hospital setting, and 127 from Hospice.

**Results:**

A high proportion of non-professional caregivers were interviewed – in both hospital (76.6%) and hospice (74.8%). There was no significant difference in the median number of days in each setting. 89.0% of hospital patients’ GPs and 85.0% of hospice patients’ GPs were interviewed. Care procedures were recorded in all hospice cases, and were missing in only 1 hospital case.52.7% of Hospital patients’ relatives and 64.12% Hospice relatives were assessed to have been caused a low level of distress through the study.

**Conclusions:**

The data shows high levels of compliance and adherence to the study instruments. This suggests that this approach to assessing quality of care is feasible, and this coupled with low levels of distress caused by the study instruments suggest effectiveness. There were no substantial differences between the hospice and hospital settings.

## Background

Research assessing the quality of care at the end of life is complicated by a number of methodological and ethical issues, and suitable methods must be found to build a strong evidence base to support the foundation of effective palliative care [1] Research focused on the dying phase (the final days and hours of life) is often found to be more problematic, and presents some very particular challenges.

Two major issues encountered in this phase concern the selection of the study population - the dying patients - and the procedures of the assessment of quality of care. Study populations may be selected either prospectively or retrospectively; the prospective approach involves the identification of an unbiased sample of dying patients over a specified period of time. The prospective identification of study participants in research in the dying phase is problematic as there are no specific criteria for identifying the dying phase [2]. Thus, retrospective selection of the study population - also called the after-death approach - has been postulated to be a suitable alternative.

The assessment of the subjective patient experience is a major challenge in researching the dying phase. The prospective approach has the benefit of potentially using the dying patient as the primary source of information, however dying patients are often the least able to participate in research [3], may either be too ill to participate or are likely to have died before they are able to participate fully [2]. Questions are also raised concerning stress placed on the patient, their capacity to answer questions fully and whether such research is a valuable use of time for patients so close to death.

In work that relies on the testimonies of informal caregivers, the identification of these individuals as such can be problematic - as an individual must self identify as being the person best informed on the deceased’s final days of life [4]. This approach is a much more reliable way of identifying a study population, but using proxy respondents exclusively may not adequately capture the subjective experience of the patient. Recall bias may also unduly influence the validity of information collected. Some studies have demonstrated that the retrospective approach is effective in evaluating concrete, observable phenomena [5], but less so in measuring the qualitative patient experience [6-8].

However, multiple assessments are possible using the retrospective approach, as the a number of persons involved with the deceased, such as family and professional staff, ancillary staff etc. can be traced through the final place of stay. This is particularly suited to the multidisciplinary nature of palliative care, where measuring the scope of care with a single scale can be difficult. Multiple measures can be amalgamated to provide an overview of the end of life experience, however this can lead to issues in identification and corroboration of significant results [9] A single outcome measuring scale is often inappropriate and runs the danger of failing to adequately assess key outcomes for patients and their families, yet a multidimensional approach needs to be justified in terms of the level of burden imposed on patients and their families, and the feasibility of using a labour intensive research approach [10].

The primary aim of this study is to evaluate the feasibility of using one particular combination of assessment methods aimed at different proxy respondents to create a means of measuring quality of care at the end of life. We also aim to explore whether there are differences in response to this approach between the hospice and hospital inpatient settings.

## Methods

### Design

This is a secondary analysis from two cluster trials. Data gathered at baseline was analysed to assess quality of care at the end of life for dying cancer patients and their families. The tools used in the original study were all aimed at retrospective analysis, after the death of the patient. In the original study, written consent was obtained from participants. The trial was approved by the ethics committee of the National Cancer Institute of Genoa (Genoa, Italy).

### The two cluster trials

In 2009 and 2010, two clinical trials were undertaken to assess the implementation of the Italian version of the Liverpool Care Pathway for the Dying Patient (LCP-I) with the aim of improving the quality of end-of-life care within hospital wards and hospices. According to the MRC Framework [11] the trials can be classified as a randomised phase III and a before/after phase II cluster trial respectively.

The trial performed in the hospital setting [12,13] was a randomised cluster trial conducted in 16 hospital wards in 5 Italian regions, where pairs of wards were allocated to receive either a modified Liverpool Care Pathway or standard end-of-life care. The quality of end-of-life care was evaluated for all eligible cancer deaths in the 3 months before the randomisation date (baseline evaluation) and in the six months after the conclusion of the LCP-I Program in the experimental ward (effectiveness evaluation). The trial performed within the hospice setting was conducted in 5 hospices in Liguria. This was a before-after study, in which the quality of end-of-life care was evaluated in the five hospices for all eligible deaths in the two months before and in the two months after the implementation of the LCP-I Program.

### Population

For the purpose of this study we used and compared baseline data from the hospital trial, and pre-intervention data from the hospice study. In the original studies, the eligibility criteria included only cancer deaths in the hospital setting, but all deaths in the hospice setting. For the purpose of this secondary analysis we excluded non-cancer deaths from the hospice sample. In both trials, deaths were excluded if the patient was a relative of a member of hospital/hospice staff.

### Setting

5 Ligurian hospices and 16 general medical wards in hospitals across five Italian regions were studied. The number of beds in the hospice setting ranged between 8–18, with between 1–5 doctors and 4–9 members of nursing staff. The number of beds in each ward in the hospital setting ranged between 24–70, with between 6–17 doctors and 15–44 members of nursing staff.

### Standard of comparison

Feasibility was measured through compliance and adherence to the study tools. In order to provide a standard to measure this against as a measure of effectiveness, we took guidance from previous studies [14] that utilised an after-death approach. To reach a level of compliance that could determine feasibility we looked to identify and make contact with at least 95% of caregivers and secure an interview with over 65%. Interviews with caregivers should have taken place between 2–4 months after the death of the patient; with face-to-face interviews comprising at least two thirds of all interviews performed. We looked for a response rate of at least 75% from GPs to requests for interview, and expected 100% of care procedures to be recorded.

### Procedures of assessment

In both settings, the procedures of assessment in the original studies were the same.

**Figure 1:**
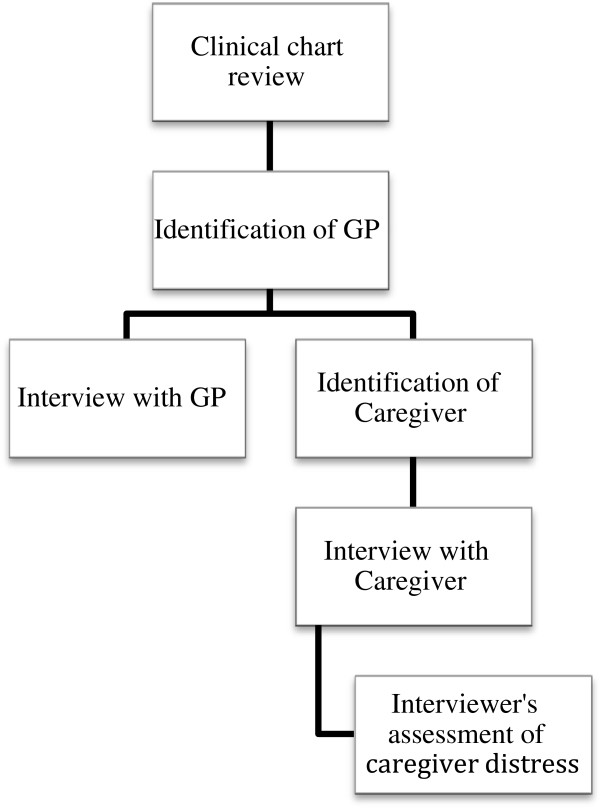
Process and sequence of data gathering.

A clinical chart review was undertaken by medical staff at the place of death, providing a record of therapeutic and medical interventions in the final three days of life. From this, the patient’s GP was contacted by researchers to answer questions on the quality of communication between themselves and the ward or hospice professionals.Both clinical charts and GPs were consulted to identify the non-professional caregiver - defined as the person who was closest to the patient during his/her last week of life in hospital or hospice. An independent interviewer, trained by the research team, contacted the identified caregiver and an interview was then arranged. The preference was for a face-to-face interview, but if this was not feasible a telephone interview took place. A professional with expertise in palliative care performed all interviews (Figure 1).

### Caregiver interview

The quality of end-of-life care provided to the patients and their families was evaluated by means of a modified version of the Toolkit After-Death Bereaved Family Member Interview [15] a semi-structured interview administered to a proxy interviewee. The interview was planned for 2–4 months after the patient’s death, though if this time scale could not be met the interview was performed outside these parameters. The interview focused on the patient’s last week of life (or less for those with shorter hospital or hospice stays). The Toolkit instrument investigates whether physical comfort and emotional support are provided to the dying patient, whether shared decision making is promoted, if care is focused on the individual, whether the needs of family members are met and if satisfactory coordination of care is achieved.

The modified version of the instrument included seven scales (1) Informing and making decisions, (2) Advance care planning, (3) Respect, dignity and kindness, (4) Family emotional support, (5) Coordination of care, (6) Family self-efficacy (7) and the Overall rating of patient-focused, family-centred care. Added to this were three symptom scales (pain, breathlessness and nausea-vomiting) from the Italian version of the ‘VOICES’ questionnaire [14] for evaluating pain, breathlessness and nausea and vomiting.

At the end of the interview, the interviewer evaluated the quality of the information provided and the emotional impact of the interview on the non-professional caregiver by responding to 4 questions, answered on a Likert scale.

### GPs interview

The quality of communication between ward or hospice professionals and patient’s GPs was evaluated by means of a semi-structured telephone interview, which took place after the patient’s death. Two questions enquired as to whether the GP was informed by the hospital/hospice about the dying phase and the death of the patient respectively. A third question explored whether the GP was aware of the terminal status of the patient.

### Clinical chart review

A standardised document for the recording of diagnostic and clinical procedures and drugs administered in the last 3 days of life was created. Nurses or physicians from the research team completed the document after the patient’s death.

### Ethics and privacy

It is important to note that the feasibility of this study is measured from the viewpoint of the Italian setting, where the study was originally situated. Differences cross-culturally in managing ethical and privacy concerns must be taken into account. The hospital cluster trial [12] received ethical approval from the Ethics Committee of the National Cancer Research Institute of Genoa (Italy) on September 14th 2009 (Reference: CCP09.001) and subsequently from the six Local Ethical Committees where the hospitals were allocated. The hospice cluster trial received ethical approval from the Ethics Committee of the National Cancer Research Institute of Genoa (Italy) on July 5^th^ 2010 (Reference: CCP10.001) and subsequently from the four Local Ethical Committees where the hospices were allocated.

Studies concerning the dying phase can be subject to particular concern from ethics committees due to the perceived vulnerability of patients and carers involved [16]. However, this particular study did not seek to elicit information from patients, and aimed to merely assess the feasibility of the investigation methods used in the studies described.

The tracing of informal caregivers through the decedent’s GP, as performed in the Italian setting, may be difficult to replicate in other countries and an alternative solution to trace relatives would need exploring.

### Data analysis

Feasibility was analysed through investigating compliance and adherence to the study instruments. The data gathered was compared between the two settings, and against the expected compliance and adherence levels derived from the PRISMA and ISDOC studies [14], which also used the after-death approach.

Compliance to the Toolkit After-Death Bereaved Family Member Interview, the ancillary 4 questions for interviewer evaluation and GP’s interviews was assessed, alongside adherence to the retrospective chart review. Adherence was also measured through assessing how closely the study instruments were utilised in accordance with the original terms suggested in the study protocol; with regard to variables such as timing and mode of interview.

The Pearson’s Chi-squared test was applied to assess heterogeneity between the samples for nominal and ordinal data, and Mann–Whitney non-parametric heterogeneity tests are to be used for continuous variables. IBM SPSS Statistics (version 19) was used for all analyses.

## Results

The two trials recruited 272 deaths (145 from hospital and 127 from hospice). Four cases were excluded because the patients were relatives of a member of staff (all from the hospital setting). In addition, four non-cancer deaths were excluded from the hospice sample.

Table 1 shows patient and care characteristics for each setting. There were demographic differences between the two populations in terms of gender – 69.0% male in hospital, and 55.1% male in hospice (P = 0.019), level of education - almost double the number of hospice patients having had over nine years of education compared to hospital patients (p = 0.005) and marital status – around a third of hospital patients classed as single, and half of hospice patients (P = 0.011) (Table 1).

**Table 1:** Demographic characteristics of cancer patients and their caregivers in the two settings

	**Hospital N = 145**		**Hospice N = 127**		**P-value**
	**n.**	**%**	**n.**	**%**	
Age (mean, range)	76	(46–96)	73	(43–96)	.238
Gender					
Male	100	69.0	70	55.1	
Female	45	31.0	57	44.9	.019
Education (years)					
9-13	21	18.3	35	36.4	
6-8	26	22.6	23	24.0	
0-5	68	59.1	38	39.6	.005
Unknown	30		31		
Marital status					
Single	42	32.3	55	48.2	
Married	88	67.7	59	51.8	.011
Unknown	15		13		
Referred from					
Home	123	87.9	51	40.2	
Nursing home	7	5.0	8	6.3	
Hospital	10	7.1	68	53.5	<.001
Unknown	5		-		
Primary tumour					
Digestive system	45	31.0	41	32.3	
Respiratory system	40	27.6	30	23.6	
Genitourinary system	14	9.7	24	18.9	
Haematological	27	18.6	7	5.5	
Breast	8	5.5	5	3.9	
Others	11	7.6	20	15.7	.003
Days in ward/hospice (median, range)	7	(1–63)	11	(1–261)	.008
**CAREGIVER**					
Age (mean, range)	55	(24–91)	54	(25–81)	.779
Gender					
Male	52	36.4	46	36.2	
Female	91	63.6	81	63.8	.981
Unknown	2				
Relationship partner	40	28.4	37	29.1	
Child	78	55.3	61	48.1	
Other	23	16.3	29	22.8	.339
Unknown	4		-		
Education (years)					
9-13	55	51.8	61	64.2	
6-8	36	34.0	28	29.5	
0-5	15	14.2	6	6.3	.101
Unknown	39		32		

Notes: Category “single” include widowed, divorced and separated.

Final stay characteristics also showed differences between hospital and hospice. A major difference between settings was where the patient was referred from, with over 90% of hospital patients having been referred from home, compared with under half of hospice patients (P = <0.001). Primary disease also had marked differences, the biggest differences being found between genitourinary tumours, under 10% of hospital patients and almost 20% of hospice and haematological tumours which comprised over 15% of hospital patients primary illness, but less than 6% of those in hospice (P = 0.003). Length of stay differed significantly between settings, with a median of 7 days in hospital and 11 in hospice (P = 0.008).

There were no significant differences in the caregiver population between settings.

A high proportion of non-professional caregivers were identified and interviewed. There were no significant differences (P = 0.630) between hospital (76.6%) and hospice (74.8%). Over 70% of caregiver interviews were performed “face to face”, with no significant difference by setting (P = 0.186). In both settings most face-to-face interviews were performed in the home of the caregiver - 56.3% of hospital caregivers and 42.1% of hospice (P = 0.029). In the hospital setting, the range of days between the death of the patient and the interview being performed was 48 – 234, in the hospice setting this was 61 – 244. There was no significant difference in the median number of days in each setting (Table 2).

**Table 2:** Characteristics of the procedures of assessment

	**Hospital N = 145**		**Hospice N = 127**		**P-value**
	**n.**	**%**	**n.**	**%**	
**Proxy interview**					
Performed interviews	111	76.6	95	74.8	
Not found	8	5.5	4	3.1	
Not present	1	0.7	-	-	
Refused	23	15.9	26	20.5	
Other	2	1.4	2	1.6	.630
Death – interview interval					
< 2 months	64	55.2	61	64.2	
2 – 4 months	38	32.8	32	33.7	
> 4 months	14	12.1	2	2.1	.023
Length of interview (minutes); median (range)	40 (10–120)		35 (15–90)		.105
Mode of interview					
Face to face	80	72.1	76	80.0	
By telephone	31	27.9	19	20.0	.186
Not performed	34		32		
Interview setting (for face to face interviews)					
Home	45	56.3	32	42.1	
Health service	17	21.2	17	22.4	
Caregiver work place	13	16.3	10	13.2	
Other	5	6.2	17	22.4	.029
**GP Interviews**					
Performed interviews	129	89.0	108	85.0	
Not found	14	9.7	14	11.0	
Not present	-	-	1	0.8	
Refused	2	1.4	1	0.8	
Other	-	-	3	2.4	.285
Death – interview interval (days); median (range)	86 (27–365)		103 (29–168)		.008
GP aware of terminal status					
Yes	89	69.5	98	90.7	
No	39	30.5	10	9.3	<.001
Unknown	17		19		
**Care procedures**					
Charts reviewed	143	98.6	127	100	.184

Notes: Unless otherwise specified n. (%).

89.0% of hospital patients’ GPs and 85.0% of hospice patients’ GPs were interviewed. A significantly (P < 0.001) higher proportion of GPs of hospice patients (90.7%) as compared to GPs of hospital patients (69.5%) were informed about the terminal phase of disease of their patients. (Table 2).

Care procedures were recorded in all hospice cases, and were missing in only 2 hospital cases.

Table 3 shows the interviewers’ assessment of whether the caregiver was well informed on the patient’s status in the last days of life. There was a similar outcome between settings, with over three quarters of hospital and hospice caregivers reported to have a high level of understanding on this subject – defined as scoring “a lot” or “completely” on the Likert scale (.108). A similar amount of respondents scored highly on comprehension of the interview questions (.915), and assessment of whether their answers were deemed to be “complete and reliable” (.127).

**Table 3:** Interviewer’s assessment of level of information of the caregivers and distress potentially caused by the interview

	**Hospital**	**Hospice**	**P-value**
	**n =110**	**n = 92**	
	**%**	**%**	
Was the caregiver well informed on the status of the patient in the last days of life?			
Not at all	NA	NA	
A little	9.1	1.1	
Somewhat	12.7	17.4	
A lot	45.5	47.8	
Completely	31.8	33.7	.108
I don’t know	0.9	-	
What is the interviewee’s level of comprehension of the questions asked in this interview?			
Not at all	-	-	
A little	1.8	2.2	
Somewhat	21.1	19.6	
A lot	39.4	40.2	
Completely	36.7	38.0	.915
I don’t know	0.9	-	
Has the caregiver provided complete and reliable answers on the patient’s situation?			
Not at all	-	-	
A little	4.6	2.2	
Somewhat	28.4	16.3	
A lot	44.0	56.5	
Completely	22.9	25.0	.127
I don’t know	-	-	
Has the administration of this interview caused distress to the caregiver?			
Not at all	12.7	16.3	
A little	40.0	47.8	
Somewhat	32.7	26.1	
A lot	9.1	6.5	
Completely	3.6	3.3	.549
I don’t know	1.8	-	

NOTE: NA is not applicable.

52.7% of Hospital patients’ relatives and 64.1% Hospice relatives were assessed by the interviewer to have been caused a low level of distress through the modified TOOLKIT interview (an answer of “not at all” of “a little” to the original question). A large proportion were judged to be “somewhat” distressed (32.7% and 26.1% respectively). However, this does leave 12.7% of the relatives of hospital patients and 9.8% of the relatives of hospice patients being judged as having a high score on the distress related scale.

### Strengths and limitations

The results show a high level of compliance and adherence to all measurement tools in each environment. Comparison between the two settings showed that, though the populations were not identical in terms of demographics and characteristics of the final stay, results according to the study instruments were comparable.

However the results do also cast light on possible problems within the study. The results concerning timing of interviews in Table 2 suggest that the ideal time frame set out in the protocol for administering interviews to caregivers and GPs deserve further investigation and consideration. Published literature calls attention to issues surrounding ideal timing of research; in order to achieve a balance between maximising the accurate recall of events, maximising the response rate and (if the respondent is a non-professional caregiver) minimising any potential distress an optimum timing must be sought. Although findings from research in this area differ, administering an interview 2–3 months after the patient death seems to be the most widely accepted guideline from the research literature [3]. Changes to this could be explored in light of reducing the circa 10% of patients in each group who scored a high level of distress on the interviewer’s assessment of the Toolkit interview.

The use of proxy respondents is a limitation intrinsic to this study and results must be interpreted with the knowledge that the main sources of information are proxies and not patients, with all that this entails.

## Discussion

This study aimed to address this issue by exploring end of life care through a multi-faceted means of assessment, as well as comparing the results of assessment between the hospital and hospice settings – comparing results against previous studies that utilised the after-death approach to assess effectiveness of the research tools. We looked to assess the feasibility of using this particular combination of research tools to provide a nuanced assessment of the end-of-life experience as assessed by proxy respondents.

Existing literature exploring the after death approach has commented on the role and reliability of proxy respondents. Proxy respondents (non-professional caregivers, GPs) are often used in research, though even when the primary caregiver is identifiable research has demonstrated that the accuracy of proxies’ evaluation can be negatively influenced by the perceived burden of caregiving [17], and that discrepancies between patients and proxies increase with their involvement in day-to day care [18,19]. On the other hand, some findings underline that proxies living with the patient are better able to report on the patient’s experience [7].

The tests in this study have shown a higher level of compliance overall than the ISDOC study that was the basis of comparison. Two thirds of interviews with caregivers were performed face to face, and the majority of those not interviewed face to face were interviewed by telephone. This study differed from ISDOC in that though the preferred interview method was face to face, the option existed to perform a telephone interview if the caregiver was to refuse the first option. This, along with allowing interviewees the freedom to choose a location for the face to face interview potentially raises the feasibility of this type of assessment by allowing interviewees to take ownership over the process, and was developed to combat non-response bias, as explored by Cassarett et al. [20].

Investigating the use of GPs as a potentially valid source of information also formed an important part of measuring the feasibility of this approach. The majority of GPs were interviewed after initial contact, and provided information on patients. Though over 30% of the GPs of hospital patients indicated that they were unaware that the patient was in the terminal phase of illness, this percentage was much lower amongst hospice GPs - who reported having this knowledge in 90.2% of cases. This information is important in planning studies that use the GP as a source of information concerning patients’ end-of-life care. The way in which GPs were used in the original study to provide details of the caregiver to be contacted would be difficult to replicate outside of Italy due to differing approaches to data protection and patient privacy. It is also important to note that this may act as a form of gatekeeping through restricting access to certain caregivers, or prioritising traditionally important family members over those who may have participated more in the caring process. Direct contact with those identified by the final place of care as being the primary informal caregiver could avoid such potential bias.

The interviewer’s evaluation of the TOOLKIT interview also helps us to understand further the concept of feasibility through providing a snapshot of the understanding of the caregiver during the interview. According to interviewer assessment, most caregivers were deemed to have a good level of information and comprehension of the patient’s condition at the end of life. In most cases the interviewers reported low or no distress caused by the interview. These levels are similar to those found by Koffman et al. [21], who also highlight the possibility that such interviews may in fact be beneficial to bereaved relatives.

Though it is widely acknowledged that a stronger evidence base is necessary to support palliative care as a complex intervention, assessing research at the end-of-life can be a difficult undertaking due to the number and depth of outcomes measured [12]. A tool that combines a number of respondents on different facets of the end-of-life experience could help to address this problem and provide a more nuanced assessment of quality of care in the dying process.

## Conclusions

The data shows high levels of compliance and adherence to all study instruments in the two settings. The combination of various forms of data collection, from various sources (caregivers, GPs, clinical documentation) and the high compliance to all forms of assessment in both hospice and hospital settings suggests that this approach for assessing information about quality of care is feasible. When combined with the interviewers’ assessment on comprehension or question, quality of answers and distress levels of interviewees results suggest that this approach is an effective combination of tools to assess quality of care at the end of life, from an after-death approach.

## Competing interests

The author(s) declare that they have no competing interests.

## Authors’ contributions

EW drafted the paper and performed the main statistical analyses. VR contributed methods and provided statistical assistance. SDL provided methodological guidance. IJH provided extensive background knowledge. GM provided theoretical expertise. MC guided the form and format of the study and writing up. All authors read and approved the final manuscript.

## Authors’ information

Collaborators: Van den Block Lieve ^a^, Meeussen Koen ^a^, Brearley Sarah ^e^, Caraceni Augusto ^g^, Cohen Joachim ^a^, Costantini Massimo ^h^, Francke Anneke ^b^, Harding Richard ^c,d^, Higginson Irene J ^c,d^, Kaasa Stein ^f^, Linden Karen ^k^, Miccinesi Guido ^i^, Onwuteaka-Philipsen Bregje ^b^, Pardon Koen ^a^, Pasman Roeline ^b^, Pautex Sophie ^j^, Payne Sheila ^e^, Deliens Luc ^a,b^

EURO IMPACT, **Euro**pean **I**ntersectorial and **M**ultidisciplinary **Pa**lliative **C**are Research **T**raining, is funded by the European Union Seventh Framework Programme (FP7/2007-2013, under grant agreement n° [264697]). EURO IMPACT aims to develop a multidisciplinary, multi-professional and inter-sectorial educational and research training framework for palliative care research in Europe. EURO IMPACT is coordinated by Prof Luc Deliens and Prof Lieve Van den Block of the End-of-Life Care Research Group, Ghent University & Vrije Universiteit Brussel, Brussels, Belgium ^a^. Other partners are: VU University Medical Center, EMGO Institute for health and care research, Amsterdam, the Netherlands ^b^; King’s College London, Cicely Saunders Institute, London ^c^, Cicely Saunders International, London ^d^, and International Observatory on End-of-Life Care, Lancaster University, Lancaster, United Kingdom ^e^; Norwegian University of Science and Technology^f^, and EAPC Research Network ^g^, Trondheim, Norway; Regional Palliative Care Network, IRCCS AOU San Martino-IST, Genoa ^h^, and Cancer Research and Prevention Institute, Florence, Italy ^i^; EUGMS European Union Geriatric Medicine Society, Geneva, Switzerland ^j^; Springer Science and Business Media, Houten, the Netherlands ^k^.

## Pre-publication history

The pre-publication history for this paper can be accessed here:

http://www.biomedcentral.com/1472-684X/13/36/prepub
